# Anomalous ellipticity dependence in nonsequential double ionization of ArXe

**DOI:** 10.1038/s41598-018-27120-x

**Published:** 2018-06-08

**Authors:** Cheng Huang, Mingmin Zhong, Zhengmao Wu

**Affiliations:** School of Physical Science and Technology, Southwest University, Chongqing, 400715 China

## Abstract

Using a three-dimensional classical ensemble method, we present a theoretical study of nonsequential double ionization of ArXe dimer aligned along the minor axis of the elliptically polarized laser pulse. Numerical results show that NSDI probability firstly increases and then decreases with the laser ellipticity increasing, which is different from atoms. Moreover, the correlated electron momentum spectra from elliptical polarization are always asymmetric, and the asymmetry is enhanced as the ellipticity increases. Analysis backward in time indicates that in NSDI of ArXe aligned along the minor axis the recollision occurs via a semi-elliptical trajectory.

## Introduction

Nonsequential double ionization (NSDI)^1,2^ is one of the most fundamental processes in intense laser-atoms/molecules interactions. It provides an excellent platform for the study of electron correlation effects^3,4^ and thus has attracted increasing experimental^5–15^ and theoretical^16–29^ efforts in the past decades. Nowadays it is accepted that NSDI occurs by an inelastic recollision process^30,31^: Firstly one electron tunnels through the suppressed potential barrier by the intense laser field and then the electron is driven back by the oscillating laser field and knocks out the second electron by colliding with the parent ion inelastically.

This recollision picture leads to the understanding that any transverse motion of the first ionizing electron is unfavourable for its return^32^. For example, in elliptically polarized laser field, the first ionizing electron is pulled away from its core by a transverse electric filed force. Generally the free electron misses its core when it returns back in longitudinal direction because of existence of a transverse deviation originating from the transverse electric field. Previous studies have indicated that the deviation originating from the transverse electric field can be compensated by a initial transverse momentum^33–35^. For the larger laser ellipticity, the transverse electric field is stronger and thus the required compensating transverse initial momentum is larger. Because the initial transverse momentum distribution is a Gaussian distribution centered at zero, NSDI yield sharply decreases with the increase of laser ellipticity^33^.

Initial transverse momentum can eliminate the transverse deviation from the transverse electric field and make recollision occur between the first ionizing electron and its core. Alternatively, if a target molecule with a large nuclear distance is positioned along the minor axis of the elliptic field, the recollision may occur between the first ionizing electron and the other core in the molecule even if the initial transverse momentum of the first ionizing electron is zero. In this case, NSDI yield may not decrease monotonously as laser ellipticity increases.

In this paper, we explore the ellipticity dependence of NSDI yield of the molecule aligned along the minor axis of the elliptic field and the electron correlation behavior. We choose ArXe dimer as the target. The equilibrium internuclear distance of ArXe dimer is about 8.0 a.u.^36^, which is comparable to the deviation from the transverse electric field. Numerical results show that NSDI yield of ArXe dimer aligned along the minor axis firstly increases and then gradually decreases with the laser ellipticity increasing, and the correlated electron momentum spectra from the elliptical polarization are asymmetric. Analysis backward in time indicates that in elliptical electric field the recollision occurs via a semi-elliptical trajectory and only the electrons ionized near the maxima of longitudinal electric field can return from negative direction and recollide with the Ar core. The single-direction return results in the asymmetry of correlated electron momentum spectrum.

## Methods

In this paper, we employ the three-dimensional (3D) classical ensemble model with a soft-core potential for the Coulomb interactions proposed by Eberly and coworkers^37,38^, which has been widely recognized as a reliable and useful approach for interpretation and prediction of strong-field double ionization phenomena^39–42^. In this model, the evolution of the two-electron system is determined by the Newton’s equations of motion (atomic units are used throughout unless stated otherwise):

1 $$\frac{{d}^{2}{\overrightarrow{r}}_{i}}{d{t}^{2}}=-\,\nabla [{V}_{ne}({\overrightarrow{r}}_{i})+{V}_{ee}({\overrightarrow{r}}_{1},{\overrightarrow{r}}_{2})]-\overrightarrow{E}(t),$$

where the subscript *i* = 1, 2 is the label of the two electrons and ${\overrightarrow{r}}_{i}$ is the coordinate of the *i*_*th*_ electron. $\overrightarrow{E}(t)=\frac{{E}_{0}f(t)}{\sqrt{1+{\varepsilon }^{2}}}[\hat{x}\,\cos (\omega t)+\hat{y}\varepsilon \,\sin (\omega t)]$ is the electric field of the elliptically polarized laser pulse. Its major axis is along x direction. *E*_0_, *ω* and *ε* are the electric field amplitude, the frequency, the ellipticity of the laser pulse, respectively. *f*(*t*) is the envelope of the 800 nm laser pulse which has a trapezoidal shape with two-cycle turn on, six cycles at full strength, and two-cycle turn off.

**Figure 1:**
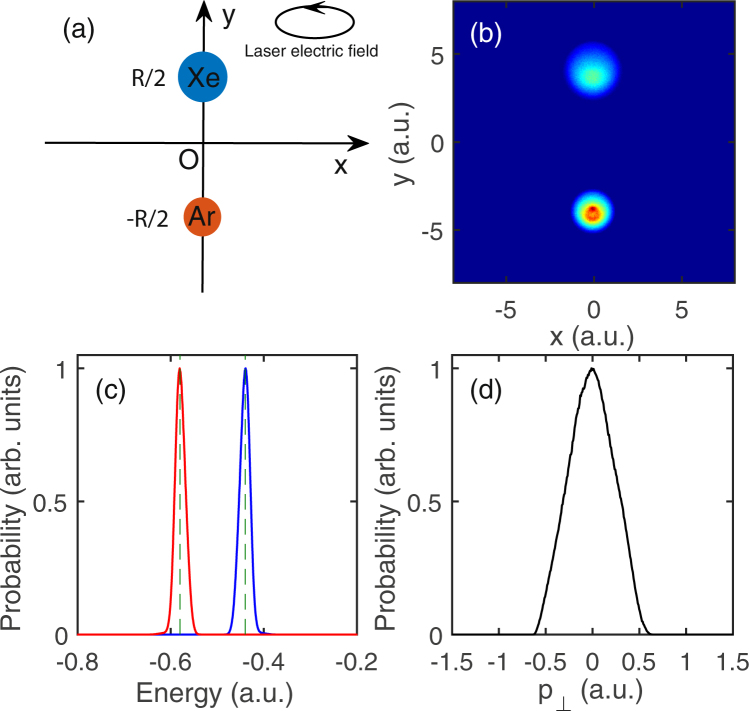
Initial state of the classical model ArXe dimer. (**a**) Sketch of ArXe dimer in the elliptically polarized laser field. *R* is the internuclear distance. (**b**) Electron distribution in x–y plane of the classical model ArXe dimer. (**c**) Initial energy distributions of electron 1 (blue) and electron 2 (red). (**d**) Initial transverse electron momentum distribution.

In this work the target is ArXe dimer aligned along the minor axis of the elliptically laser field (i.e., y axis), as shown in Fig. 1(a). Its equilibrium internuclear distance is R = 8.0 a.u. The Ar and Xe are fixed at (0, −*R*/2,0) and (0, *R*/2,0) respectively. Here we are focused on NSDI induced by heteronuclear recollision, which corresponds to ionization channel ArXe → Ar^+^ + Xe^+^ + 2*e*. Thus we only need to consider one outmost electron of Xe and Ar respectively. For convenience the electron around Xe is denoted electron 1 and the electron around Ar is denoted electron 2. The interaction between electron and the parent ion can be modeled by the two-center soft-core Coulomb potential ${V}_{ne}({\overrightarrow{r}}_{i})$  =  $-\,1/\sqrt{{x}_{i}^{2}+{({y}_{i}-R/2)}^{2}+{z}_{i}^{2}+{a}_{1}}-1/\sqrt{{x}_{i}^{2}+{({y}_{i}+R/2)}^{2}+{z}_{i}^{2}+{a}_{2}}$. a_1_ and a_2_ is the soft parameters of Xe^+^ and Ar^+^, which make the model molecule avoid unphysical autoionization. In our calculation we have set *a*_1_ = 4.0 and *a*_2_ = 2.0. The electron-electron interaction is ${V}_{ee}({\overrightarrow{r}}_{1},{\overrightarrow{r}}_{2})$  =  $1/\sqrt{{({\overrightarrow{r}}_{1}-{\overrightarrow{r}}_{2})}^{2}+b}$. b is set 0.01 to prevent numerical singularity.

To obtain the initial condition, firstly electron 1 and electron 2 are put respectively near Xe^+^ and Ar^+^ and their positions are obtained by using a random Gaussian distribution. Once the positions of electron 1 and electron 2 are given, their potential energies are fixed. In order to match the first ionization energies of Xe (0.44 a.u.) and Ar (0.58 a.u.), we need to add a specific kinetic energy to electron 1 and electron 2 respectively. According to the kinetic energies of electron 1 and electron 2, the amplitudes of the momentum vectors of the two electrons can be obtained. Here the directions of the momentum vectors of the two electrons are assigned randomly. Then two electrons are allowed to evolve a sufficiently long time (400 a.u.) in the absence of the laser field to obtain stable position and momentum distribution. The electron position distribution of the initial ensemble is shown in Fig. 1(b). The energy distributions of electron 1 (blue curve) and electron 2 (red curve) are shown in Fig. 1(c). Their energy distributions peak at −0.44 a.u. and −0.58 a.u. respectively, which match well the first ionization energies of Xe and Ar. The electron transverse momentum distribution of the initial ensemble is shown in Fig. 1(d). Once the initial ensemble is obtained, the laser field is turned on and all trajectories are evolved in the combined Coulomb and laser fields. We check the energies of the two electrons at the end of the laser field, and a DI event is determined if the energies of both electrons are positive, where the energy of each electron contains the kinetic energy, potential energy of the electron-ion interaction, and half electron-electron repulsion.

## Results

### Ellipticity dependence of NSDI probability

**Figure 2:**
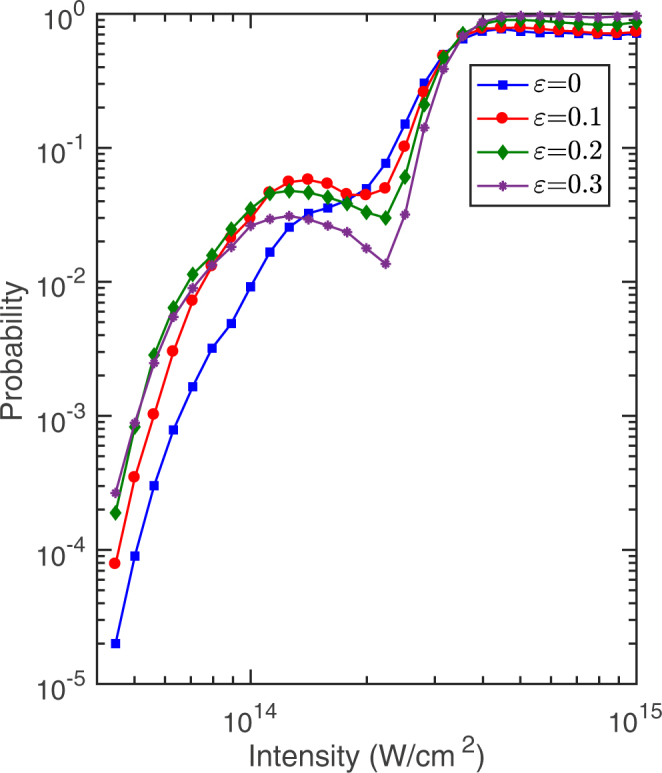
Probabilities of double ionization as a function of the intensity of the 800 nm laser pulse for the ellipticity 0, 0.1, 0.2 and 0.3.

Figure 2 shows the probability of double ionization as a function of the intensity of the 800 nm laser pulse for the ellipticity 0(blue), 0.1(red), 0.2(green), 0.3(purple). One can see that all of the four intensity-dependent curves exhibit a clear knee structure which is recognized as the signature of strong-field NSDI. In this work we are focused on NSDI of ArXe dimer, i.e., the knee region. It is obvious that NSDI probability of *ε* = 0 (linear polarization) is not the largest. This is different from atoms where NSDI probability of linear polarization is larger than that of elliptical polarization. More interestingly, the four curves cross each other. It means that the ellipticity where NSDI probability is the largest changes with the variation of the laser intensity. For example, for 1.2 × 10^14^ W/cm^2^ the NSDI probability of *ε* = 0.1 is larger than those of *ε* = 0.2, 0.3 and 0. For 9 × 10^13^ W/cm^2^ the NSDI probability of *ε* = 0.2 is slightly larger than those of the other three ellipticities.

**Figure 3:**
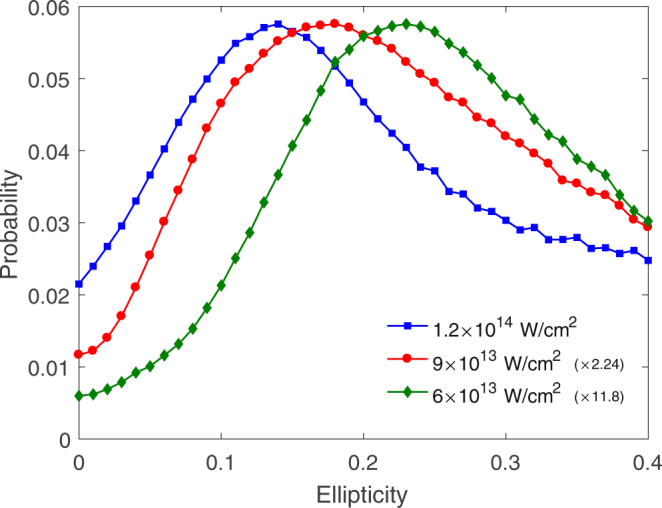
NSDI probabilities as a function of the ellipticity for the laser intensity 6 × 10^13^ W/cm^2^ (green), 9 × 10^13^ W/cm^2^ (red) and 1.2 × 10^14^ W/cm^2^ (blue).

In order to more clearly exhibit the ellipticity dependence of NSDI yield of ArXe by elliptically polarized laser pulses, in Fig. 3 we present NSDI probability as a function of the ellipticity for the laser intensities 6 × 10^13^ W/cm^2^ (green), 9 × 10^13^ W/cm^2^ (red) and 1.2 × 10^14^ W/cm^2^ (blue). For the convenience of comparison, those values from the laser intensities 6 × 10^13^ W/cm^2^ (green) and 9 × 10^13^ W/cm^2^ are multiplied the factors 11.8 and 2.24 respectively. It is obvious that NSDI probability always firstly increases and then decreases and reaches a maximum at a critical ellipticity. The location of the maximum, i.e., the critical ellipticity, is significantly different for different laser intensities. The critical ellipticity gradually decreases with the laser intensity increasing. At 6 × 10^13^ W/cm^2^, the critical ellipticity is 0.23. When the laser intensity reaches 9 × 10^13^ W/cm^2^ (red) and 1.2 × 10^14^ W/cm^2^, it becomes 0.18 and 0.14.

### Semi-elliptical recollision trajectories

**Figure 4:**
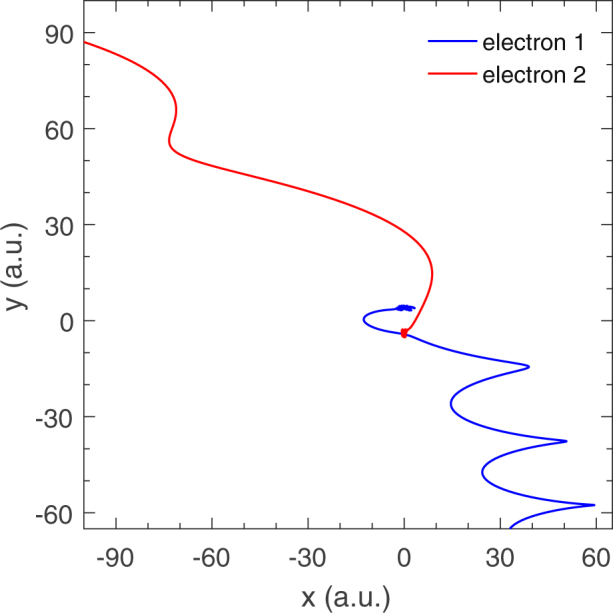
A typical recollision trajectory from those NSDI events at the ellipticity 0.18 and intensity 9 × 10^13^ W/cm^2^.

Because in our calculation the propagation of the electrons in time is classical, we can tag individual key trajectories and then carefully back examine their histories at the end of a simulation. Here, by tracing those classical NSDI trajectories, we find that for ArXe recollision occurs via semi-elliptical trajectories. It is different from atoms in elliptically polarized electric fields, where the returning electron need execute one or several ellipses before recollision^34^. It is because for atoms the first ionization and recollision occur at the same core and the electron has to travel one or several whole ellipses to achieve recollision. However, for ArXe dimer the first ionization and recollision occur at different cores and thus a whole ellipse motion is not required to recollision. This is clearly shown in Fig. 4 by a typical recollision trajectory from those NSDI events at *ε* = 0.18 and I = 9 × 10^13^ W/cm^2^. Firstly the outmost electron 1 of Xe with smaller ionization energy is ionized and the electron 2 around Ar is still bound. In major axis direction (x direction), electron 1 is first pulled away from Xe core and then is driven back when the electric field reverses. In minor axis direction (y direction), electron 1 is accelerated by the transverse electric field and moves to −y direction. When electron 1 arrives around 0 in x direction and −4 a.u. in y direction, i.e., the position of Ar core, recollision occurs and electron 2 is knocked out. In this process the first ionizing electron only moves in −x part and experiences half an ellipse, not a whole ellipse, before recollision. So we call it semi-elliptical recollision trajectories.

## Discussion

In order to obtain more deep understanding for ultrafast dynamics in NSDI of ArXe by the elliptically polarized laser pulse and its dependence on the laser ellipticity, we perform statistical analysis for classical NSDI trajectories and find out the single ionization time t_*SI*_, the recollision time t_*r*_, and the double ionization time t_*DI*_. Here, the single ionization time is defined as the instant when one electron achieves positive energy or passes over the suppressed potential barrier^43^. The double ionization time is defined as the instant when both electrons achieve positive energies^44^. The recollision time is defined as the instant of closest approach of the two electrons after the first ionizing electron escapes the core.

**Figure 5:**
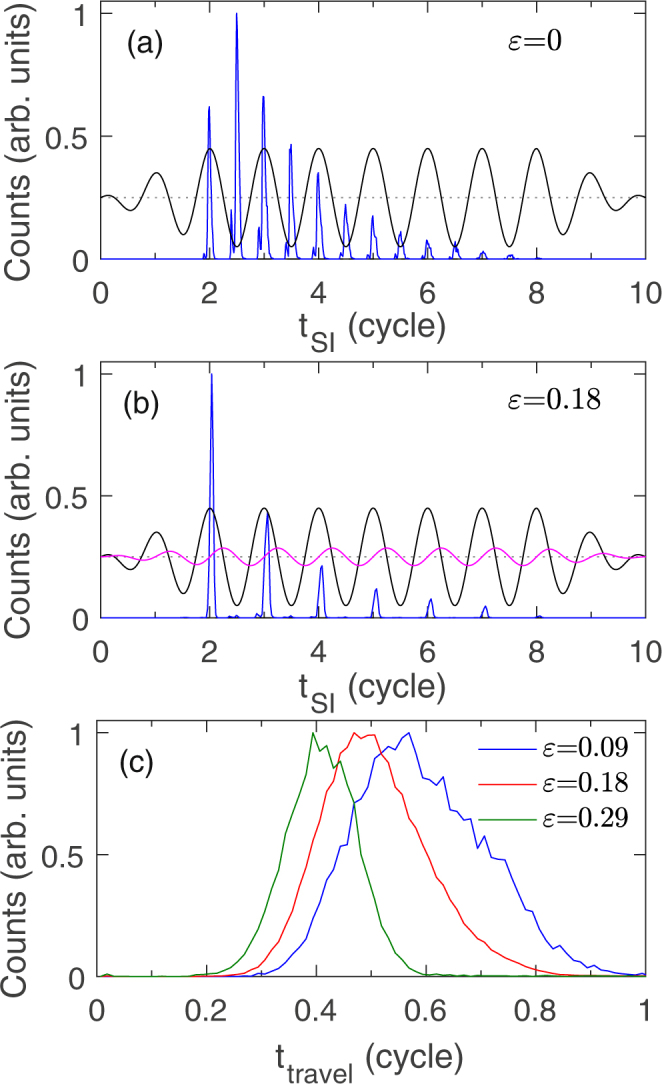
(**a**,**b**) Show distributions of the single ionization time (t_*SI*_) for those NSDI events from *ε* = 0 and 0.18. The black and magenta curves show the electric field in x and y directions respectively. (**c**) Shows distribution of the travel time (t_*r*_ − t_*SI*_) for those NSDI events from *ε* = 0.09, 0.18 and 0.29. The laser intensity is 9 × 10^13^ W/cm^2^.

Figure 5(a,b) show the distributions of the single ionization time for those NSDI events from *ε* = 0 and 0.18 at the laser intensity of 9 × 10^13^ W/cm^2^. The black and magenta curves show the electric field in x and y directions respectively. The zero of the electric field is marked by a black dashed line. The most obvious difference between *ε* = 0 and 0.18 is that for *ε* = 0 the single ionization occurs near each extremum of the longitudinal electric field (E_*x*_), but for *ε* = 0.18 the single ionization only occurs near those maxima of E_*x*_. It means that for linearly polarized laser field the ionizing electrons from each extremum of E_*x*_ can induce double ionization but for elliptically polarized laser field only those ionizing electrons from the maxima of E_*x*_ can induce double ionization and those from the minima of E_*x*_ can not. This can be understood as follows. Because of the smaller ionization energy for Xe than Ar, the first ionizing electron is from Xe. For linearly polarized laser fields, after single ionization the free electron is only driven by the electric field in x direction. The occurrence of recollision needs a negative transverse initial momentum to make the first ionizing electron move from the vicinity of Xe (y = 4 a.u.) to the vicinity of Ar (y = −4 a.u.). The situation is the same for those electrons emitted to −x and +x direction. Thus the electrons ionized from the maxima and minima of E_*x*_ can induce the same number of NSDI events. However, for elliptically polarized laser fields after single ionization the free electron is also driven by the transverse electric field E_*y*_. If the electron is ionized near the maximum of E_*x*_, the subsequent transverse electric field E_*y*_ is positive [see the magenta curve in Fig. 5(b)] and can pull the ionizing electron to Ar core, which facilitates recollision. In contrast, if the electron is ionized near the minimum of E_*x*_, the subsequent transverse electric field E_*y*_ is negative [see the magenta curve in Fig. 5(b)] and will drive the ionizing electron to +y direction. It makes the electron move away from Ar core and the recollision can not occur.

For linearly polarized laser fields, a negative transverse initial momentum is required for the occurrence of recollision in NSDI of ArXe. For elliptically polarized laser fields, the transverse electric field can pull the first ionizing electron to the vicinity of Ar core in y direction. When the ellipticity is small, the transverse electric field is not enough strong to drive the first ionizing electron to arrive at the Ar core. In this case a negative transverse initial momentum is still necessary for recollision. With the ellipticity increasing the required initial transverse momentum becomes small and thus NSDI probability gradually increases. When the ellipticity is large enough, the transverse electric field can drive the first ionizing electron to the Ar core. If the ellipticity further increases, the transverse electric field becomes larger and thus the time that the first ionizing electron is pulled from the Xe core to the Ar core becomes short [see Fig. 5(c)]. The shorter travel time results in the smaller returning energy. Thus for the large ellipticity the NSDI probability decreases with the ellipticity increasing. As a whole, the NSDI probability firstly increases and then decreases with the ellipticity increasing.

The discussion above has indicated that the transverse motion of the first ionizing electron is determined by the transverse electric field E_*y*_. For the elliptically polarized laser pulse E_*y*_ is proportional to the laser intensity and the ellipticity. If the laser intensity increases the transverse electric field of the laser pulse with smaller ellipticity can drive the first ionizing electron from the Xe core (y = 4 a.u.) to the Ar core (−4 a.u.). Thus the critical ellipticity gradually decreases with the laser intensity increasing.

We can make a rough estimate of the critical ellipticity which corresponds to the largest probability of NSDI for a given intensity based on the simple-man model. In this model, the electron is considered to be located at the Xe core (x = 0, y = 4 a.u.) with zero initial velocity when the first ionization happens. Then it is only governed by the laser electric field. As is well known, for a linearly polarized laser field the trajectory where the first electron is ionized at 0.048 cycle after the extremum of the electric field and returns at 0.709 cycle has the highest returning energy. In order to make recollision occur for this type of trajectory in the ArXe system driven by the elliptically polarized laser field, the transverse distance the electron moves during the travel time (from 0.048 cycle to 0.709 cycle after the extremum of the field) should be equal to 8 a.u. (the nuclear distance of ArXe). According to this condition, we can calculate the amplitude of the required transverse electric field. It is 0.0048 a.u. This transverse electric field corresponds to different ellipticities for different laser intensities. For 6 × 10^13^ W/cm^2^ (green), 9 × 10^13^ W/cm^2^ (red) and 1.2 × 10^14^ W/cm^2^ used in this work, the corresponding ellipticities are respectively 0.117, 0.095 and 0.082. Firstly, these estimated values gradually decrease with the laser intensity increasing, which well agrees with our ensemble calculation. Secondly, these estimated values are smaller than those values from our ensemble calculation. It could be attributed to two factors. One is the effect of Coulomb potential from the parent ion which is ignored in the simple-man model. The other is the contribution of the short trajectory where the travel time of the electron is smaller than that of the particular trajectory with the highest returning energy. Our statistics show there are a large number of short trajectories contributing to NSDI.

**Figure 6:**
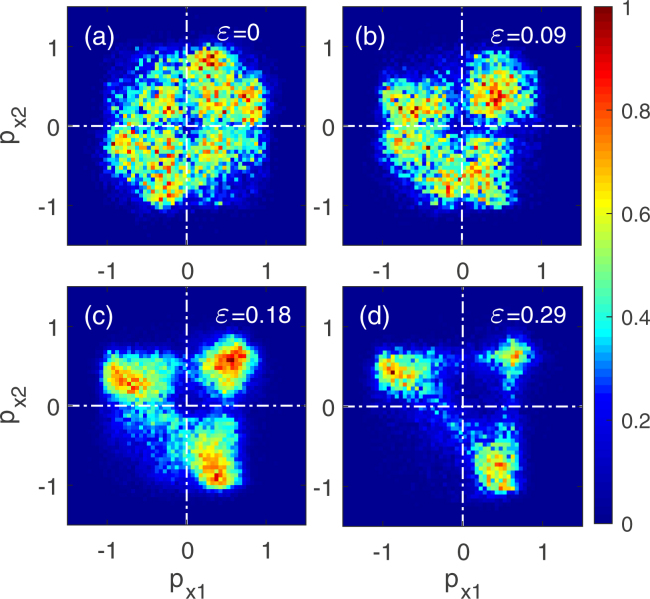
Correlated electron momentum spectra along the major axis direction of the elliptically polarized laser pulse at the intensity of 9 × 10^13^ W/cm^2^ for the ellipticity *ε* = 0 (**a**), 0.09 (**b**), 0.18 (**c**) and 0.29 (**d**). The two electrons are not distinguished.

Now we turn to the ellipticity dependence of the electron correlation behavior. Figure 6 shows the correlated electron momentum spectra along the major axis direction (x direction) of the elliptically polarized laser pulse. The laser intensity is 9 × 10^13^ W/cm^2^. The ellipticities are 0, 0.09, 0.18 and 0.29 for panel (a) to (d). The two electrons are not distinguished. These correlated electron momentum spectra have notable difference. Firstly, for linear polarization case (*ε* = 0) shown in Fig. 6(a), the electron pairs almost uniformly distribute in four quadrants. However, for elliptically polarized laser pulses (*ε* ≠ 0) those distributions are not uniform. They are asymmetric with respect to the minor diagonal. It is because for linear polarization the electrons ionized from the maximum and minimum of E_*x*_ have the same probability to induce recollision, but for elliptical polarization only the electrons ionized from the maximum of E_*x*_ can return to the vicinity of Ar core from −x direction and induce recollision. The single-direction recollision results in the asymmetry of the correlated electron momentum spectrum from elliptical polarization.

Furthermore, the population of NSDI electron pairs in the third quadrant decreases with the ellipticity increasing, as shown in Fig. 6(b–d). When the ellipticity increases to 0.29, there are almost no electron pairs left in the third quadrant. Those NSDI events in the third quadrant need that both electrons involved in NSDI have negative longitudinal momentum (x direction). Our statistics find that electron 1 (the first ionizing electron from Xe) more likely drifts out with a positive final longitudinal momentum than with a negative longitudinal momentum with the ellipticity increasing. The decrease of the probability of electron 1 with negative final longitudinal momentum results in the population of NSDI electron pairs in the third quadrant decreases with the ellipticity increasing.

**Figure 7:**
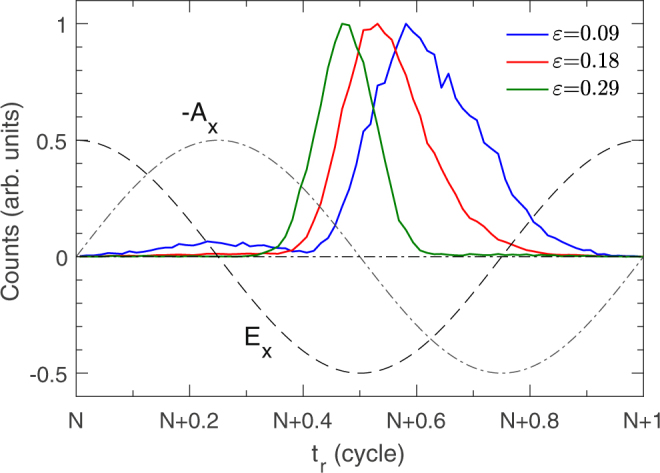
Distribution of the recollision time for the intensity 9 × 10^13^ W/cm^2^ and the ellipticity 0.09, 0.18 and 0.29. The dashed and dashdot curves show the electric field (E_*x*_) and negative vector potential (−A_*x*_) in x direction.

The outmost electron 1 of Xe is emitted around the maximum of E_*x*_ [see Fig. 5(b)]. Firstly, it moves to −x direction in a positive longitudinal electric field, and then is driven back from −x direction when the electric field reverses. Thus it has a positive longitudinal momentum before recollision. After recollision, electron 1 transfers a part of energy to electron 2 and it remains free with a positive longitudinal momentum. If the effect of Coulomb potential of the parent ion is ignored, the final longitudinal momentum of electron 1 is *p*_*fx*_ = *p*_*ix*_ − *A*_*x*_(*t*_*r*_) where *p*_*ix*_ is the residual longitudinal momentum after recollision and *A*_*x*_ is the vector potential of the electric field in x direction. −A_*x*_(*t*_*r*_) is the acceleration from the longitudinal electric field after recollision. After recollision electron 1 has a positive residual longitudinal momentum *p*_*ix*_. In order to make the final longitudinal momentum *p*_*fx*_ of electron 1 negative, a large enough acceleration to −x direction is required. Figure 7 shows the distribution of the recollision time for the ellipticity 0.09 (blue), 0.18 (red) and 0.29 (green). The sketch of the electric field (E_*x*_) and negative vector potential (−A_*x*_) in x direction is also shown. The acceleration from the electric field to electron 1 in −x direction increases with increasing time delay between the recollision and the minimum of E_*x*_. As shown in Fig. 7, with the ellipticity increasing, the recollision time moves to the minimum of E_*x*_. It is because the successful recollision needs shorter travel time for the larger ellipticity, which has been discussed above. For the larger ellipticity electron 1 will obtain a smaller acceleration to −x direction, which is difficult to pull back the returning electron 1 and make it finally drift to −x direction. Thus the probability of electron 1 with negative final longitudinal momentum decreases with the ellipticity increasing. Finally it results in the decrease of the population of the electron pairs in the third quadrant of the correlated electron momentum spectrum with the increase of the laser ellipticity.

## Conclusion

To conclude, we have investigated the ellipticity dependence of NSDI of ArXe dimer aligned along the minor axis of the elliptically polarized laser field. Different from atoms in elliptical electric field, where NSDI probability monotonously decreases with the ellipticity increasing, NSDI probability of ArXe dimer aligned along the minor axis firstly increases and then decreases and reaches a maximum at a critical ellipticity. Moreover, the critical ellipticity gradually decreases with the laser intensity increasing. Analysis backward in time indicates that in NSDI of ArXe dimer aligned along the minor axis the recollision occurs via a semi-elliptical trajectory, and with the ellipticity increasing the travel time of the first ionizing electron shortens and the recollision moves to the extrema of the longitudinal electric field. It results in the decrease of the population of the electron pairs in the third quadrant of the correlated electron momentum spectrum as the ellipticity increases.

## References

[1] Fittingoff DN, Bolton PR, Chang B, Kulander KC (1992). Observation of nonsequential double ionization of helium with optical tunneling. Phys. Rev. Lett..

[2] Walker B (1994). Precision measurement of strong field double ionization of helium. Phys. Rev. Lett..

[3] Figueira de Morisson Faria C, Liu X (2011). Electron-electron correlation in strong laser fields. J. Mod. Opt..

[4] Becker W, Liu X, Jo Ho P, Eberly JH (2012). Theories of photoelectron correlation in laser-driven multiple atomic ionization. Rev. Mod. Phys..

[5] Weber Th (2000). Correlated electron emmision in multiphoton double ionization. Nature.

[6] Staudte A (2007). Binary and recoil collisions in strong field double ionization of Helium. Phys. Rev. Lett..

[7] Rudenko A (2007). Correlated two-electron momentum spectra for strong-field nonsequential double ionization of He at 800 nm. Phys. Rev. Lett..

[8] Bergues B (2012). Attosecond tracing of correlated electron-emission in non-sequential double ionization. Nature Commun..

[9] Camus N (2012). Attosecond correlated dynamics of two electrons passing through a transition state. Phys. Rev. Lett..

[10] Zhang L (2014). Subcycle control of electron-electron correlation in double ionization. Phys. Rev. Lett..

[11] Liu Y (2014). Strong-field double ionization through sequential release from double excitation with subsequent coulomb scattering. Phys. Rev. Lett..

[12] Wolter B (2015). Strong-field physics with mid-IR Fields. Phys. Rev. X.

[13] Gong X (2015). Channel-resolved above-threshold double ionization of acetylene. Phys. Rev. Lett..

[14] Wang Y (2016). Recoil-ion momentum distribution for nonsequential double ionization of Xe in intense midinfrared laser fields. Phys. Rev. A.

[15] Eckart S (2016). Nonsequential double ionization by counterrotating circularly polarized two-color laser fields. Phys. Rev. Lett..

[16] Lein M, Gross EKU, Engel V (2000). Intense-field double ionization of helium: identifying the mechanism. Phys. Rev. Lett..

[17] Feuerstein B (2001). Separation of recollision mechanisms in nonsequential strong field double ionization of Ar: the role of excitation tunneling. Phys. Rev. Lett..

[18] Emmanouilidou A, Tchitchekova DS (2011). Strongly driven molecules: Traces of soft recollisions for intermediate intensities in the over-the-barrier regime. Phys. Rev. A.

[19] Hao X (2014). Quantum Effects in Double Ionization of Argon below the Threshold Intensity. Phys. Rev. Lett..

[20] Parker JS (2006). High-energy cutoff in the spectrum of strong-field nonsequential double ionization. Phys. Rev. Lett..

[21] Chen Z, Liang Y, Lin CD (2010). Quantum theory of recollisional (e, 2e) process in strong field nonsequential double ionization of helium. Phys. Rev. Lett..

[22] Ye D (2015). Scaling laws of the two-electron sum-energy spectrum in strong-field double ionization. Phys. Rev. Lett..

[23] Maxwell AS, Figueira de Morisson Faria C (2016). Controlling Below-Threshold Nonsequential Double Ionization via Quantum Interference. Phys. Rev. Lett..

[24] Chaloupka JL, Hickstein DD (2016). Dynamics of strong-field double ionization in two-color counterrotating fields. Phys. Rev. Lett..

[25] Mancuso CA (2016). Controlling nonsequential double ionization in two-color circularly polarized femtosecond laser fields. Phys. Rev. Lett..

[26] Ben S, Wang T, Xu T, Guo J, Liu X (2016). Nonsequential double ionization channels control of Ar with few-cycle elliptically polarized laser pulse by carrier-envelope-phase. Opt. Express.

[27] Li Y (2016). Nonsequential double ionization with mid-infrared laser fields. Sci. Rep.

[28] Ruiz C, Plaja L, Roso L, Becker A (2006). Ab initio calculation of the double ionization of helium in a few-cycle laser pulse beyond the one-dimensional approximation. Phys. Rev. Lett..

[29] Ma X, Li M, Zhou Y, Lu P (2017). Nonsequential double ionization of Xe by mid-infrared laser pulses. Opt. Quant. Electron.

[30] Corkum PB (1993). Plasma perspective on strong-field multiphoton ionization. Phys. Rev. Lett..

[31] Schafer KJ, Yang B, DiMauro LF, Kulander KC (1993). Above threshold ionization beyond the high harmonic cutoff. Phys. Rev. Lett..

[32] Guo C, Li M, Nibarger JP, Gibson GN (1998). Single and double ionization of diatomic molecules in strong laser fields. Phys. Rev. A.

[33] Wang X, Eberly JH (2010). Elliptical polarization and probability of double ionization. Phys. Rev. Lett..

[34] Wang X, Eberly JH (2010). Elliptical Trajectories in Nonsequential Double Ionization. New J. Phys..

[35] Kamor A, Mauger F, Chandre C, Uzer T (2013). How Key Periodic Orbits Drive Recollisions in a Circularly Polarized Laser Field. Phys. Rev. Lett..

[36] Wu J (2013). Probing the tunnelling site of electrons in strong field enhanced ionization of molecules. Nature Commun.

[37] Panfili R, Eberly JH, Haan SL (2001). Comparing classical and quantum simulations of strong-field double-ionization. Opt. Express.

[38] Haan SL, Breen L, Karim A, Eberly JH (2006). Variable time lag and backward ejection in full-dimensional analysis of strong-field double ionization. Phys. Rev. Lett..

[39] Dong S, Zhang Z, Bai L, Zhang J (2015). Scaling law of nonsequential double ionization. Phys. Rev. A.

[40] Huang C, Guo W, Zhou Y, Wu Z (2016). Role of coulomb repulsion in correlated-electron emission from a doubly excited state in nonsequential double ionization of molecules. Phys. Rev. A.

[41] Chen J, Nam CH (2002). Ion momentum distributions for He single and double ionization in strong laser fields. Phys. Rev. A.

[42] Huang C, Zhong M, Wu Z (2016). Recollision dynamics in nonsequential double ionization of atoms by long-wavelength pulses. Opt. Express.

[43] Haan SL, Smith ZS, Shomsky KN, Plantinga WP (2008). Anticorrelated electrons from weak recollisions in nonsequential double ionization. J. Phys. B.

[44] Zhou Y (2017). Dissection of electron correlation in strong-field sequential double ionization using a classical model. Opt. Express.

